# IFNγ-Stimulated B Cells Inhibit T Follicular Helper Cells and Protect Against Atherosclerosis

**DOI:** 10.3389/fcvm.2022.781436

**Published:** 2022-02-02

**Authors:** Hidde Douna, J. de Mol, Jacob Amersfoort, Frank H. Schaftenaar, Mate G. Kiss, Bianca E. Suur, Mara J. Kroner, Christoph J. Binder, Ilze Bot, Gijs H. M. Van Puijvelde, Johan Kuiper, Amanda C. Foks

**Affiliations:** ^1^Division of BioTherapeutics, Leiden Academic Centre for Drug Research (LACDR), Leiden University, Leiden, Netherlands; ^2^Department of Laboratory Medicine, Medical University of Vienna, Vienna, Austria; ^3^CeMM Research Center for Molecular Medicine of the Austrian Academy of Sciences, Vienna, Austria

**Keywords:** cardiovascular disease, atherosclerosis, B cells, PD-L1, interferon-gamma

## Abstract

B and T cells are interconnected in the T follicular helper—germinal center B cell (T_FH_-GC B cell) axis, which is hyperactive during atherosclerosis development and loss of control along this axis results in exacerbated atherosclerosis. Inhibition of the T_FH_–GC B cell axis can be achieved by providing negative co-stimulation to T_FH_ cells through the PD-1/PD-L1 pathway. Therefore, we investigated a novel therapeutic strategy using PD-L1-expressing B cells to inhibit atherosclerosis. We found that IFNγ-stimulated B cells significantly enhanced PD-L1 expression and limited T_FH_ cell development. To determine whether IFNγ-B cells can reduce collar-induced atherosclerosis, *apoE*^−/−^ mice fed a Western-type diet were treated with PBS, B cells or IFNγ-B cells for a total of 5 weeks following collar placement. IFNγ-B cells significantly increased PD-L1^hi^ GC B cells and reduced plasmablasts. Interestingly, IFNγ-B cells–treated mice show increased atheroprotective Tregs and T cell-derived IL-10. In line with these findings, we observed a significant reduction in total lesion volume in carotid arteries of IFNγ-B cells-treated mice compared to PBS-treated mice and a similar trend was observed compared to B cell-treated mice. In conclusion, our data show that IFNγ-stimulated B cells strongly upregulate PD-L1, inhibit T_FH_ cell responses and protect against atherosclerosis.

## Introduction

Cardiovascular disease (CVD) remains a major global health problem despite great developments in diagnosis and treatment. The underlying cause for CVD is atherosclerosis, which is a chronic autoimmune-like disease and is characterized by the formation of lipid-rich lesions in the arteries. The current available treatments are aimed at lipid lowering and lead to a 25–30% relative risk reduction, indicative of an urgent need for novel disease-modifying drugs. In the last decade, accumulating evidence identified the immune system as a major contributor to the pathology of atherosclerosis (1). For this reason, considerable effort has been devoted to restore the dysregulation of the immune system and inflammatory pathways in atherosclerosis.

B and T-lymphocyte dependent immune responses play a key role in the pathophysiology of atherosclerosis and the role of most B and T cell subsets in atherosclerosis is now well-defined. For instance, B1 cells have shown a consistent atheroprotective effect (2–5), while in multiple studies B2 cells were seen to contribute to atherosclerosis (6–9). In addition, the involvement of Th1, Th2 and Treg cells in atherosclerosis has also been examined comprehensively as reviewed in (10). In contrast, only recently the contribution of other leukocyte subpopulations such as follicular (FO) B cells, marginal zone (MZ) B cells and follicular helper T cells (T_FH_) to atherosclerosis have been identified. Although FO B cells, MZ B cells and T_FH_ cells are radically different cell types, they appear to be interconnected in the T_FH_–germinal center (GC) B cell axis. FO B cells enter germinal centers, subsequently undergo proliferation and isotype switching and can differentiate in short-lived plasmablasts, which can further differentiate into long-lived plasma cells or memory cells. FO B cells promote the recruitment of T_FH_ cells and the generation of germinal centers through expression of inducible co-stimulator ligand (11). Previously, it has been shown that the T_FH_–GC B cell axis is hyperactivated and promotes lesion formation in both apolipoprotein E-deficient (*apoE*^−/−^) mice (12) and low-density lipoprotein receptor-deficient (*ldlr*^−/−^) mice fed a hypercholesterolemia-inducing diet (13). In addition, both T_FH_ cells and FO B cells are proatherogenic and can aggravate atherosclerosis (14, 15).

The T_FH_–GC B cell axis can be regulated by the co-inhibitory programmed death-1 (PD-1)/PD-L1 pathway. T_FH_ cells highly express PD-1 and their accumulation can be controlled by MZ B cells which express PD-L1. In response to high cholesterol levels, MZ B cells upregulate the expression of PD-L1 and thereby regulate T_FH_ cell accumulation which limits an exacerbated adaptive immune response (13). However, this mechanism fails to completely arrest disease development. Interestingly, in autoimmune encephalomyelitis, it has been shown that adoptive transfer of B cells expressing high levels of PD-L1 limited disease severity (16). Whether PD-L1 expressing B cells can also be used therapeutically to inhibit atherosclerosis development has not yet been reported. In this study, we therefore induced PD-L1-expressing B cells and investigated whether adoptive transfer of these cells could inhibit atherosclerosis development.

## Materials and Methods

### Animals

All animal work was approved by the Leiden University Animal Ethics Committee and the animal experiments were performed conform the guidelines from Directive 2010/63/EU of the European Parliament on the protection of animals used for scientific purposes. Female C57BL/6, B6.SJL-PtprcaPepcb/BoyCrl (also known as CD45.1) and apolipoprotein E-deficient (*apoE*^−/−^) mice were bred in house and kept under standard laboratory conditions. Diet and water were provided *ad libitum*. All injections were administered i.v. to the lateral tail vein in a total volume of 100 μl. During the experiments, mice were weighed, and blood samples were obtained by tail vein bleeding. At the end of experiments, mice were anesthetized by a subcutaneous injection of a cocktail containing ketamine (40 mg/ml), atropine (0.1 mg/ml), and xylazine (8 mg/ml). Mice were bled and perfused with phosphate-buffered saline (PBS) through the left cardiac ventricle.

### Cell Culture

B cells were isolated from splenocytes of C57BL/6 or *apoE*^−/−^ mice using CD19^+^ microbeads (Miltenyi Biotec) and cultured in complete RPMI medium. Isolated B cells were cultured with different concentrations of heat killed *Staphylococcus aureus* (Invivogen), B-cell activating factor (BAFF; R&D systems) or interferon-gamma (IFNγ; ThermoFisher) for 24 h. For co-culture experiments, CD4^+^ T cells were isolated from splenocytes of C57BL/6 mice using a CD4^+^ T cell isolation kit (Miltenyi Biotec). For some experiments, the CD4^+^ T cells were labeled using a CellTrace Violet Cell Proliferation kit (ThermoFisher) according to the instructions of the manufacturer. B and T cells were co-cultured and stimulated with an agonistic plate-bound CD3 antibody (5 μg/ml) for 72 h. For adoptive transfer experiments, B cells were isolated from splenocytes of *apoE*^−/−^ or CD45.1 mice, cultured in RPMI medium and stimulated for 24 h with 20 ng/ml of IFNγ. Subsequently, cells were washed, checked for purity using flow cytometry and resuspended for injections with PBS. For the injection of untouched B cells, B cells were freshly isolated from splenocytes of *apoE*^−/−^ or CD45.1 mice and directly used for adoptive transfer experiments.

### Real-Time Quantitative PCR

RNA was extracted from cultured B cells by using Trizol reagent according to manufacturer's instructions (Invitrogen) after which cDNA was generated using RevertAid M-MuLV reverse transcriptase according to the instructions of the manufacturer (Thermo Scientific). Quantitative gene expression analysis was performed using Power SYBR Green Master Mix on a 7500 Fast Real-Time PCR system (Applied Biosystems). Gene expression was normalized to housekeeping genes. Primer sequences are available in Supplementary Table 1.

### *In vivo* Experiments

For all *in vivo* experiments, *apoE*^−/−^ mice were used. *ApoE*^−/−^ mice were fed a Western-type diet (WTD) containing 0.25% cholesterol and 15% cacao butter (SDS, Sussex, UK). For the pilot study, mice were pre-fed a WTD for 2 weeks and were subsequently treated with 2 × 10^6^ freshly isolated or cultured B cells and sacrificed after 3 days. To assess the impact of WTD on the percentage of PD-L1^hi^ B cells, *apoE*^−/−^ mice were fed a chow diet or a WTD for 2 or 7 weeks. For atherosclerosis experiments, carotid artery plaque formation was induced after 2 weeks of WTD feeding by perivascular collar placement in these mice as described previously (17). Mice continued to be fed a WTD for 5 weeks and during this period received 3 injections with either freshly isolated B cells (2 × 10^6^ cells/injection), B cells stimulated with 20 ng/ml IFNγ for 24 h (2 × 10^6^ cells/injection) or PBS. To discriminate between adoptively transferred cells and endogenous cells, B cells were isolated from CD45.1 mice for the last injection. Time between injections was 2 weeks. At the end of experiments, mice were sacrificed and relevant organs were harvested for analysis.

### Cytokine Analysis

Isolated splenocytes from PBS, B cell or IFNγ-B cell treated mice were cultured in complete RPMI medium and stimulated for 72 h with anti-CD3 (1 μg/ml) and anti-CD28 (0.5 μg/ml). The levels of cytokines in culture supernatants were measured using a Luminex bead-based multiplex assay (ProcartaPlex, Thermo Fisher Scientific) on a Luminex Instrument (MAGPIX). Recombinant cytokine standards (Thermo Fisher Scientific) were used to calculate cytokine concentrations and data were analyzed using Bio-Rad software.

### Flow Cytometry

For flow cytometry analysis, Fc receptors of single cell suspensions were blocked with an unconjugated antibody against CD16/CD32. Samples were then stained with a fixable viability marker (ThermoScientific) to select live cells. Next, cells were stained with anti-mouse fluorochrome-conjugated antibodies (Supplementary Table 2). Regular flow cytometry was performed on a Cytoflex S (Beckman Coulter) and the acquired data were analyzed using FlowJo software. Gates were set according to isotype or fluorescence minus one controls.

### Serum Measurements

Serum was acquired by centrifugation and stored at −20°C until further use. Total serum titers of IgM, IgG1, IgG2c and oxidized LDL-specific antibodies were quantified by ELISA as previously described (5).

### Histology

Carotid arteries and hearts were frozen in OCT compound (TissueTek) and stored at −80°C until further use. Transverse cryosections proximal to the collar were collected and mounted on Superfrost adhesion slides (ThermoFisher). To determine lesion size, cryosections were stained with hematoxylin and eosin (Sigma-Aldrich). Quantification of lesion size was assessed every 100 μm from the first section with visible lesion proximal to the collar until no lesion could be observed. Plaque volume was determined with lesion size and the total distance of the lesions in the carotid artery. Phenotypic analysis of the lesion was performed on sections containing the largest three lesions. Collagen content in the lesion was assessed with a Masson's trichrome staining according to the manufacturers protocol (Sigma-Aldrich). Necrotic core size was determined manually by selecting acellular areas in the Masson's trichrome stained sections and shown as absolute area and percentage of the total plaque area. Corresponding sections on separate slides were also stained for monocyte/macrophage content using a monoclonal rat IgG2b antibody (MOMA-2, 1:1000, AbD Serotec) followed by a goat anti-rat IgG-horseradish peroxidase antibody (1:100, Sigma-Aldrich) and color development using the ImmPACT NovaRED substrate (Vector Laboratories). For the detection of vascular smooth muscle cells, cryosections were stained with a monoclonal rat alpha-smooth muscle actin antibody conjugated to Alexa fluor 647 (1:1500, Novus Biologicals). Furthermore, cryosections were stained with a monoclonal rat CD4 antibody conjugated to FITC (1:150, eBioscience) and a monoclonal rat IgG2b isotype control conjugated to FITC (1:150, MBL) to determine CD4^+^ T cell infiltration. For all fluorescent images, cryosections were blocked with αCD16/32 Fc block (1:250, Biolegend) and nuclei were stained with DAPI. All slides were analyzed with a Leica DM-RE microscope and LeicaQwin software (Leica Imaging Systems).

### Statistics

All data are expressed as mean ± SEM. Data were tested for significance using a Student's *t*-test for two normally distributed groups. Data from three groups or more were analyzed by an ordinary one-way ANOVA test followed by Holm-Sidak *post-hoc* test. Probability values of *p* < 0.05 were considered significant. All statistical analyses were performed using GraphPad Prism.

## Results

### IFNγ-Stimulated B Cells Express High Levels of PD-L1

**Figure 1:**
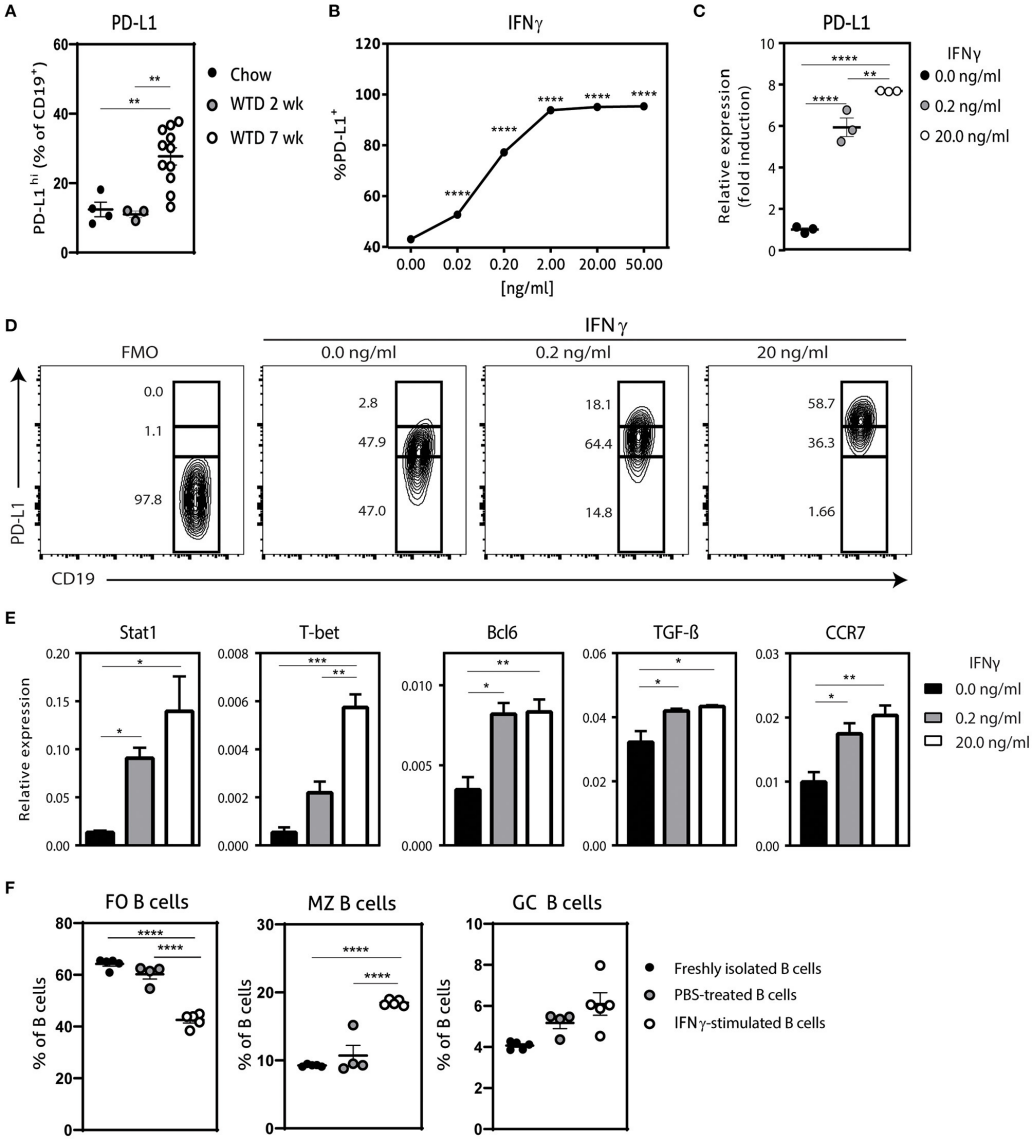
Characterization of IFNγ-stimulated B cells. **(A)** PD-L1^hi^ expressing CD19^+^ B cells were determined in spleens of *apoE*^−/−^ mice fed a regular Chow diet or Western type diet for 2 or 7 weeks using flow cytometry. **(B)** CD19^+^ B cells were isolated from C57BL/6 mice and stimulated for 24 h with different doses interferon-gamma (IFNγ) after which PD-L1 protein expression was measured with flow cytometry. **(C)** CD19^+^ B cells were unstimulated or stimulated with 0.2 ng/ml or 20.0 ng/ml IFNγ for 24 h, after which mRNA expression of PD-L1 was assessed using qPCR. **(D)** B cells as stimulated in (C) were analyzed for PD-L1^lo^, PD-L1^int^ and PD-L1^hi^ expression with flow cytometry. **(E)** mRNA expression was analyzed for depicted genes in B cells as stimulated in **(C)**. **(F)** FO B cells (CD23^+^) MZ B cells (CD23^−^ CD21^+^) and GC B cells (GL-7^+^ CD95+) were determined in CD19^+^ B cells stimulated with 20.0 ng/ml IFNγ for 24 h. Data are analyzed with a One-Way ANOVA and shown as mean ± SEM (^*^p < 0.05, ^**^p < 0.01, ^***^p < 0.001, ^****^p < 0.00001). *n* = 3–11/group.

Previously, it has been shown that hypercholesterolemia promotes PD-L1 expression on B cells in *ldlr*^−/−^ mice, which can regulate T_FH_ cell accumulation, limiting an exacerbated adaptive immune response (13). In order to investigate if the administration of a Western-type diet (WTD) also affects PD-L1-expressing B cells in *apoE*^−/−^ mice, we compared PD-L1 expression on B cells from chow fed *apoE*^−/−^ mice and *apoE*^−/−^ mice fed a WTD for 2 and 7 weeks using flow cytometry (Figure 1A; Supplementary Figure 1A). Whereas we observed no significant differences in PD-L1^hi^ B cells between chow fed *apoE*^−/−^ mice and *apoE*^−/−^ mice fed a WTD for 2 weeks, longer administration of WTD increases the percentage of PD-L1^hi^ B cells, in line with previous findings. Nonetheless, this elevation in PD-L1 expressing B cells upon hypercholesterolemia is not sufficient to halt disease development (13), indicating the need to further stimulate the regulation of the T_FH_–GC B cell axis through the PD-1/PD-L1 pathway. We therefore aimed to generate a population of PD-L1^hi^ B cells *ex vivo*, to adoptively transfer in WTD fed *apoE*^−/−^ mice to halt atherosclerosis. We explored several stimuli that previously have been shown to control PD-L1 expression (18, 19) (Supplementary Figures 1B,C; Figure 1) and found that IFNγ dose-dependently increased the number of PD-L1^+^ B cells, with 20.0 ng/ml of IFNγ leading to an almost pure population of PD-L1-expressing B cells (Figure 1B). Using qPCR, we also found an almost eight-fold induction of PD-L1 on mRNA level after IFNγ stimulation (Figure 1C). Furthermore, we observed that the majority of IFNγ-stimulated B cells expressed very high levels of PD-L1 (Figure 1D). Previously, it has been shown that IFNγ-signaling in B cells drives STAT-1 dependent expression of T-bet and BCL-6 and switches them toward a GC B cell phenotype with increased IFNγ and IL-6 production (20, 21). In line with this, our *ex vivo* IFNγ-stimulated B cells (IFNγ-B cells) showed a similar increase in STAT1, T-bet and BCL-6 (Figure 1E) gene expression. In contrast, we observed a trend toward less IL-6 and no difference in IFNγ expression (Supplementary Figure 1D). Interestingly, IFNγ stimulation resulted in a strong significant increase in TGF-β expression (Figure 1E). We also investigated the chemokine receptor profile of these B cells and found a dose-dependent increase in CCR7 expression (Figure 1E), while there was no effect on CXCR5 or Ebi-2 (Supplementary Figure 1D). This change in chemokine receptor expression is typical of B cells that migrate toward the T-B cell border in lymphoid tissues in response to the CCL21 gradient (22). Hence, these data indicate that IFNγ-stimulated B cells express high levels of PD-L1 and TGF-β and a chemokine profile that homes B cells to the T-B cell border. In line with these findings, we observed that IFNγ stimulation of B cells not only induced coinhibitory PD-L1 expression on all B cells (Figure 1C) but also increased the percentage of GC B cells and MZ B cells (Figure 1F). Together with a decrease in FO B cells, we thus show that IFNγ stimulation generates a B cell population with an enhanced anti-inflammatory phenotype.

### IFNγ-Stimulated B Cells Inhibit T_FH_ Cells *in vitro* and *in vivo*

**Figure 2:**
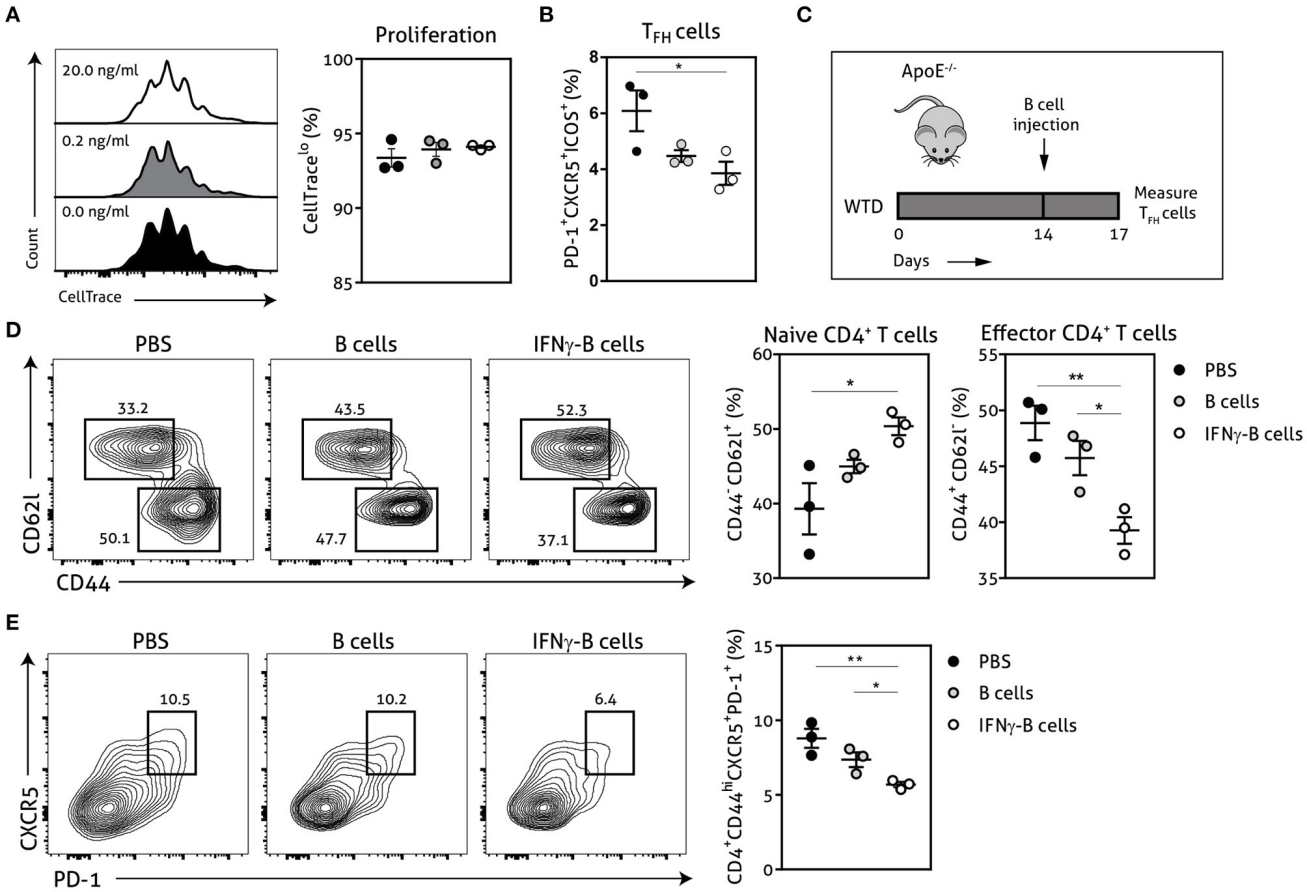
IFNγ-B cells inhibit T follicular helper cells *in vitro* and *in vivo*. Isolated CD19^+^ B cells were unstimulated or stimulated with 0.2 ng/ml or 20.0 ng/ml IFNγ for 24 h and co-cultured with isolated CD4^+^ T cells. The coculture was stimulated with anti-CD3 (5 μg/ml) for 72 h after which **(A)** proliferation of CD4^+^ T cells was analyzed with CellTrace Violet and **(B)** the number of follicular CD4^+^ T helper (T_FH_) cells were analyzed. **(C)** *apoE*^−/−^ mice were fed a Western type diet for 2 weeks after which they either received PBS, freshly isolated CD19^+^ B cells (B cells) or CD19^+^ B cells stimulated with 20.0 ng/ml IFNγ for 24 h (IFNγ-B cells). After three days, mice were sacrificed and spleens were isolated for flow cytometry analysis for **(D)** naive (CD62l^+^CD44^−^) and effector (CD62l^−^CD44^hi^) CD4^+^ T cells and **(E)** T follicular helper cells (CD4^+^CD44^hi^CXCR5^+^PD-1^+^). Representative flow charts of the CD4^+^ T cell population are shown. Data are analyzed with a One-Way ANOVA and shown as mean ± SEM (*p < 0.05, **p < 0.01). *n* = 3/group.

Next, we explored the functional effects of IFNγ-stimulated B cells on T_FH_ cell development using a CD4^+^ T cell and B cell coculture. We stimulated wild-type CD4^+^ T cells for 72 h with anti-CD3 in the presence of unstimulated or IFNγ-stimulated *apoE*^−/−^ B cells. Although we did not observe any difference in proliferative capacity of CD4^+^ T cells (Figure 2A), we found a remarkable decrease of T_FH_ cells when CD4^+^ T cells were cocultured with IFNγ-stimulated B cells compared to unstimulated B cells (Figure 2B). These findings illustrate that IFNγ-B cells are able to curb T_FH_ cell development *in vitro*. We subsequently tested IFNγ-stimulated B cells in an *in vivo* setting by adoptively transferring either freshly isolated B cells or B cells stimulated with 20.0 ng/ml of IFNγ for 24 h into *apoE*^−/−^ mice. To induce initial T_FH_ cell accumulation, mice were fed a Western-type diet for 2 weeks before they received the adoptive transfers (Figure 2C) (13). We observed a remarkable reduction in effector CD4^+^ T cells in mice treated with IFNγ-B cells compared to mice that received PBS or B cells (Figure 2D). Contrary, the number of naïve CD4^+^ T cells was increased in IFNγ-B cells treated mice compared to mice receiving PBS (Figure 2D). Most importantly, treatment of mice with IFNγ-B cells resulted in a strong reduction in T_FH_ cells compared to mice that were administered with PBS or B cells (Figure 2E). These data demonstrate that IFNγ-B cells are also able to inhibit T_FH_ cells *in vivo*.

### Adoptive Transfer of IFNγ-B Cells During Atherosclerosis Development Affects the T_FH_–GC B Cell Axis

**Figure 3:**
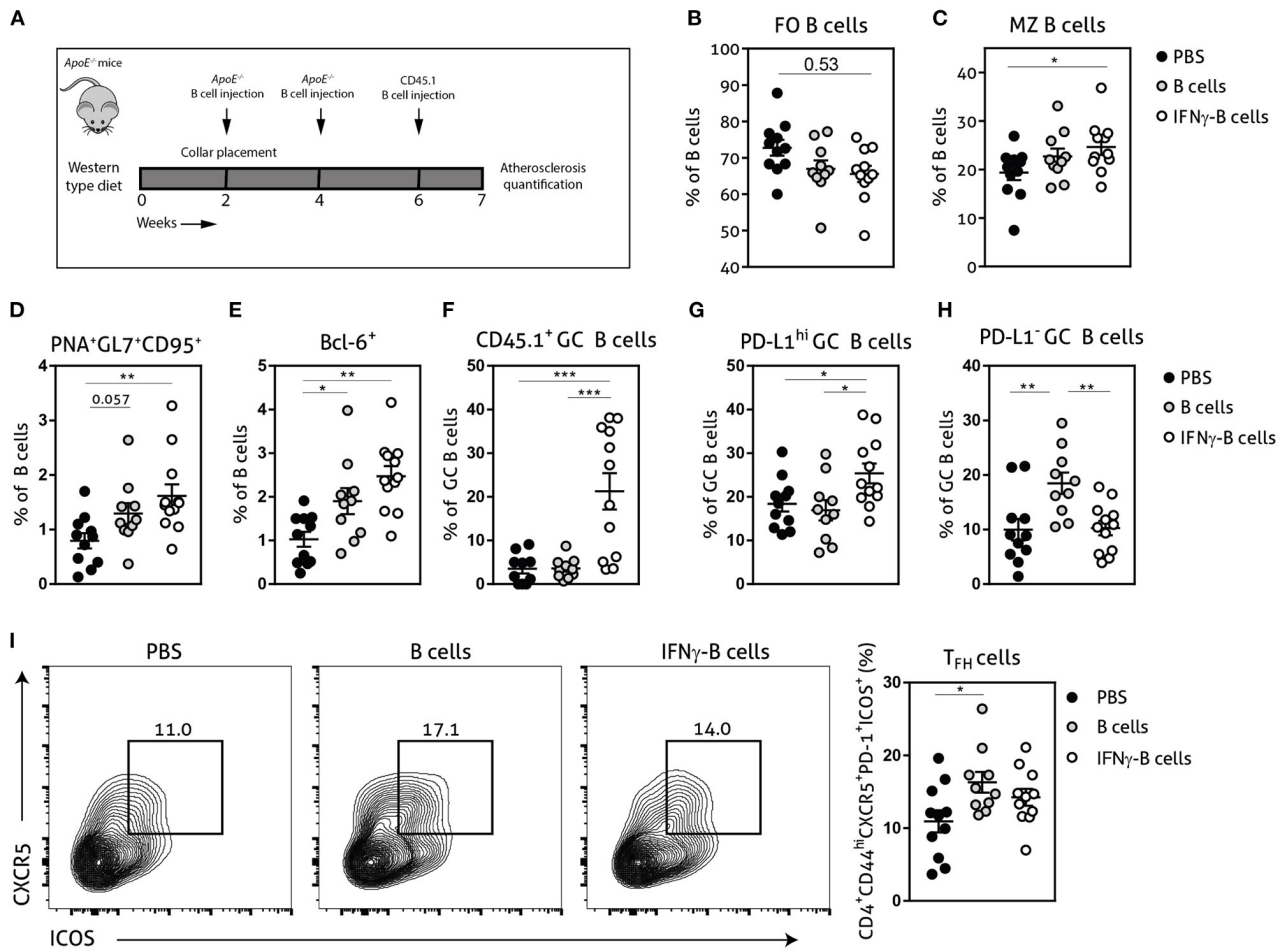
Adoptive transfer of IFNγ-B cells during atherosclerosis development affects the T_FH_–GC B cell axis. **(A)** *ApoE*^−/−^ mice were fed a Western type diet for 7 weeks. After 2 weeks they received a perivascular collar and were treated with PBS, freshly isolated B cells (B cells) or B cells stimulated with 20.0 ng/ml IFNγ for 24 h (IFNγ-B cells). Mice received a total of three injections and injections were spaced every two weeks. After 7 weeks, mice were sacrificed and spleens were analyzed with flow cytometry. Flow cytometry quantification of **(B)** follicular (FO) B cells, **(C)** marginal zone (MZ) B cells, **(D)** extracellular staining of germinal center B cells, **(E)** intracellular staining of germinal center B cells, **(F)** CD45.1^+^ germinal center B cells, **(G)** PD-L1^hi^ germinal center B cells, **(H)** PD-L1^−^ germinal center B cells and **(I)** flow charts and quantification of follicular helper T cells (CD44^hi^PD-1^+^CXCR5^+^ICOS^+^). Data are analyzed with a One-Way ANOVA and shown as mean ± SEM (*p < 0.05, **p < 0.01, ***p < 0.001). *n* = 10–12/group.

Given the *in vitro* and *in vivo* regulatory effects of IFNγ-B cells on T_FH_ cells, we further investigated whether these B cells would be able to restrict T_FH_ cell numbers during atherosclerosis development. We fed *apoE*^−/−^ mice a Western-type diet for 2 weeks after which we placed a perivascular collar and started treatment with either PBS, B cells or IFNγ-B cells (Figure 3A). After a total of 3 injections and 7 weeks of Western-type diet, we harvested the organs and analyzed the immune cells associated with the T_FH_–GC B cell axis. IFNγ-B cell-treated mice showed a trend toward reduced FO B cells (Figure 3B) and a significant increase in MZ B cells compared to mice receiving PBS (Figure 3C). Moreover, mice that received IFNγ-B cells showed a significant increase in the number of GC B cells compared to mice that were administered PBS (Figures 3D,E). By using the CD45 congenic marker system, we showed that adoptively transferred IFNγ-B cells indeed reached the germinal center (Figure 3F) and locally increased the number of PD-L1^hi^ B cells (Figure 3G). As shown in Figures 3D,E, freshly isolated B cells also increased the number of GC B cells, but these were not derived from the adoptive transfer (Figure 3F) and were PD-L1^−^ (Figure 3H). At sacrifice, we did not observe a difference in T_FH_ cells in IFNγ-B cell-treated mice, while adoptive transfer of B cells resulted in an increase in T_FH_ cells compared to PBS-treated mice (Figure 3I).

**Figure 4:**
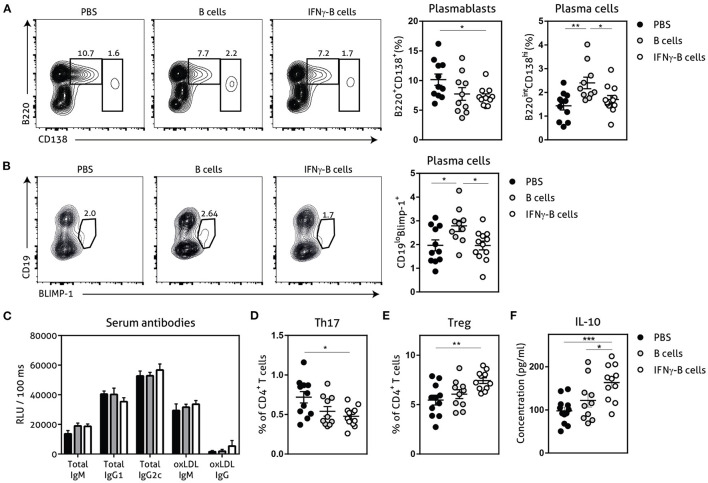
Effects of adoptive transfer of IFNγ-B cells on the humoral immunity. *ApoE*^−/−^ mice were fed a Western type diet for 7 weeks. After 2 weeks they received a perivascular collar and were treated with PBS, freshly isolated B cells (B cells) or B cells stimulated with 20.0 ng/ml IFNγ for 24 h (IFNγ-B cells). Mice received a total of three injections and injections were spaced every two weeks. After 7 weeks, mice were sacrificed and spleens were analyzed with flow cytometry. **(A)** Flow charts and quantification of plasmablasts (B220^+^CD138^+^) and plasma cells (B220^lo^CD138^hi^). **(B)** Flow charts and quantification of CD19^lo^BLIMP-1^+^ plasma cells. **(C)** Serum was analyzed for circulating antibodies by ELISA. Spleens were analyzed with flow cytometry for **(D)** Th17 cells (RORyt^+^) or **(E)** Treg cells (FoxP3^+^). **(F)** Splenocytes were isolated and stimulated with anti-CD3 (1 μg/ml) and anti-CD28 (0.5 μg/ml) for 72 h after which supernatant was collected and analyzed for cytokine expression with a multiplex analysis. Quantification of IL-10 concentration is shown. Data are analyzed with a One-Way ANOVA and shown as mean ± SEM (*p < 0.05, **p < 0.01). *n* = 10–12/group, ***p < 0.001.

Next, we assessed the number of plasmablasts and plasma cells and found a significant reduction in plasmablasts when mice received IFNγ-B cells compared to mice receiving PBS (Figure 4A). Mice that received B cells showed a similar trend toward less plasmablasts and also a significant increase in plasma cells compared to PBS- and IFNγ-B cells-treated mice. Since BLIMP-1 is the driving transcription factor for plasma cell generation (23), we also measured BLIMP-1 expression which revealed a significant increase in BLIMP-1^+^ cells in mice that received B cells compared to mice receiving PBS or IFNγ-B cells (Figure 4B). Since plasmablasts and plasma cells are responsible for the humoral immunity, we measured circulating antibodies. However, neither B cell treatments led to a significant difference in circulating antibodies (Figure 4C).

### Adoptive Transfer of IFNγ-B Cells Promotes an Anti-inflammatory CD4^+^ T Cell Response

The CD4^+^ T cell response is also highly involved in the pathogenesis of atherosclerosis and PD-L1^hi^ B cells have previously shown to restrict CD4^+^ T cell differentiation (16). While we did not observe any differences in Th1 or Th2 CD4^+^ T cells between mice treated with IFNγ-B cells, B cells or PBS in our study (Supplementary Figure 2), we observed a significant decrease in Th17 cells (Figure 4D) and a significant increase in atheroprotective regulatory T cells (Figure 4E; Tregs) in mice treated with IFNγ-B cells. We next measured cytokine levels of *ex vivo* anti-CD3 and anti-CD28 stimulated splenocytes for 72 h with a multiplex assay. In line with the increase in Tregs, we observed a significant increase in IL-10 production by splenocytes from IFNγ-B cells treated mice compared to splenocytes from mice treated with PBS or B cells (Figure 4F).

### Adoptive Transfer of IFNγ-B Protects Against Lesion Formation

**Figure 5:**
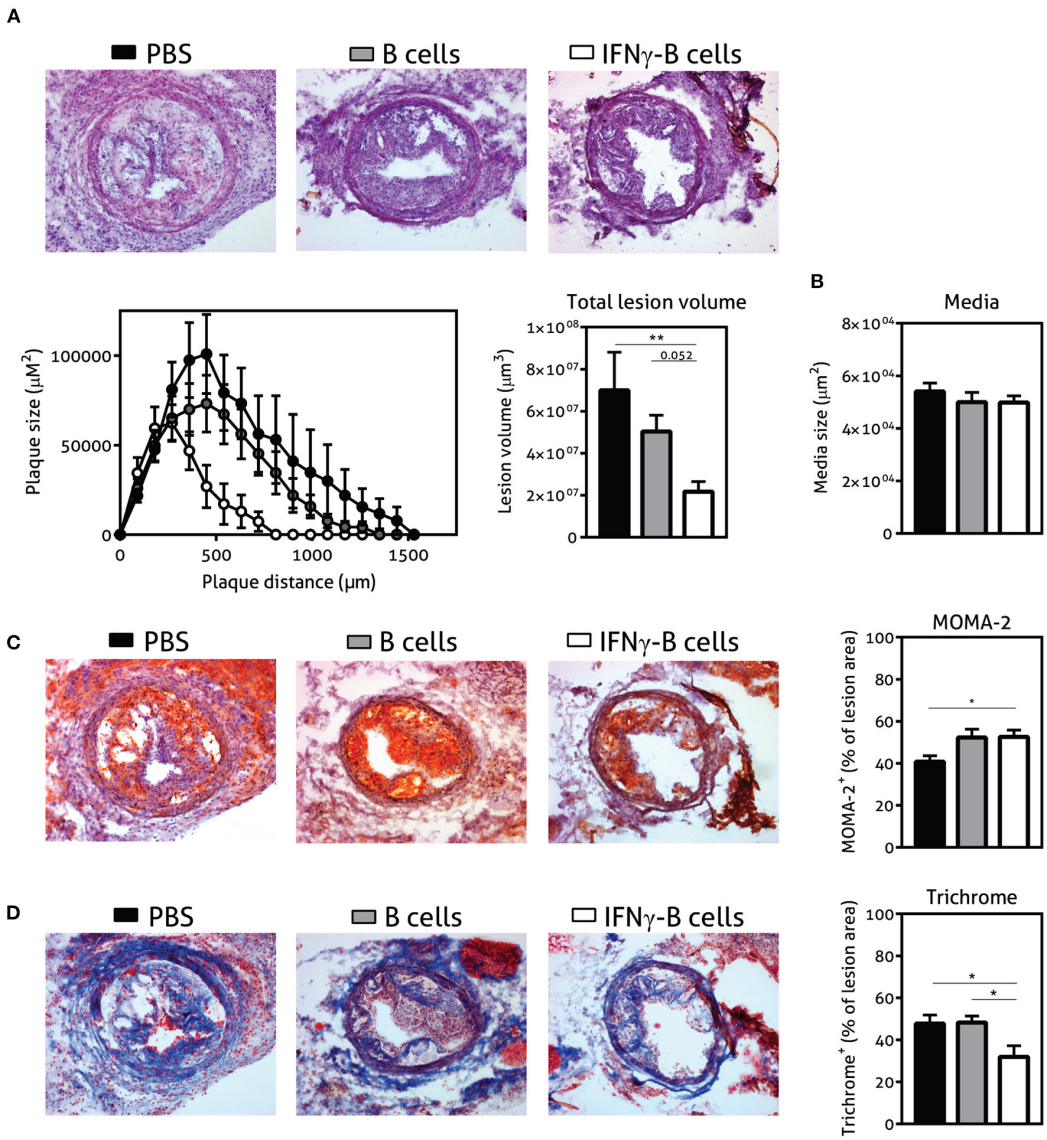
Adoptive transfer of IFNγ-B protects against atherosclerosis. *ApoE*^−/−^ mice were fed a Western type diet for 7 weeks. After 2 weeks they received a perivascular collar and were treated with PBS, freshly isolated B cells (B cells) or B cells stimulated with 20.0 ng/ml IFNγ for 24 h (IFNγ-B cells). Mice received a total of three injections and injections were spaced every two weeks. After 7 weeks, mice were sacrificed and **(A)** total lesion volume and **(B)** media size was determined in the right carotid artery with a hematoxylin and eosin staining. Lesion phenotype was determined in sections containing the largest lesions. **(C)** Macrophage content was analyzed using a MOMA-2 staining. **(D)** Collagen content was quantified with a Trichrome staining. Data are analyzed with a One-Way ANOVA and shown as mean ± SEM (*p < 0.05, **p < 0.01). *n* = 8–12/group.

Given the immune-regulating effects of IFNγ-B cells, we subsequently assessed whether adoptive transfer of these IFNγ-B cells was able to reduce collar-induced atherosclerosis in *apoE*^−/−^ mice. We quantified lesion development in the carotid arteries and found a significant reduction in total lesion volume when mice were treated with IFNγ-B cells compared to PBS and a similar trend was found when compared to B cell-treated mice (Figure 5A). Media size did not differ between the treatment groups (Figure 5B). Furthermore, there were no differences in weight gain and serum cholesterol levels (Supplementary Figures 3A,B). We also assessed lesion phenotype at the site of the maximal lesion. This revealed that mice treated with IFNγ-B cells showed an early lesion phenotype with relatively more macrophages than collagen compared to mice that received B cells or PBS (Figures 5C,D), suggesting that adoptive transfer of IFNγ-B cells inhibited atherosclerotic lesion progression toward more advanced lesions. Evaluation of vascular smooth muscle cells (VSMCs) using α-smooth muscle actin did not reveal any differences (Supplementary Figure 4A) and we also did not observe altered necrotic core formation within the atherosclerotic plaque in IFNγ-B cell treated mice compared to the other groups (Supplementary Figure 4B). Finally, we did not observe significant differences in CD4^+^ T cell infiltration between the treatment groups (Supplementary Figure 4C) and only very low numbers of CD4^+^ T cells were found, suggesting that the reduction in lesion size is caused by systemic anti-atherogenic effects of PD-L1^hi^ B cells, rather than a local effect.

## Discussion

A proatherogenic role has been reported for the T_FH_–GC B cell axis (12–14). Direct depletion of T_FH_ cells in *ldlr*^−/−^ mice resulted in a reduction of atherosclerosis (14), and loss of control on the T_FH_–GC B axis by depletion of MZ B cells (13) or CD8^+^ regulatory T cells (12) aggravated atherosclerosis. We now show that adoptive transfer of PD-L1 expressing B cells inhibit T_FH_ cell responses both *in vitro* and *in vivo* and protect against atherosclerosis.

Using *ex vivo* stimulation of B cells with IFNγ we rapidly provided a large and almost pure population of PD-L1-expressing B cells. IFNγ is an extensively investigated cytokine with a broad range of immune actions. Interestingly, while we show here that IFNγ stimulation of B cells results in a regulatory phenotype that reduces the number of T_FH_ cells, earlier work identified a role for IFNγ in the generation of spontaneous germinal centers and B cell autoreactivity (20, 24). These previous data were, however, mainly acquired in the context of autoimmunity, where B cells receive a multitude of different signals and IFNγ signaling seems primarily to synergize with BCR-, CD40- and TLR-mediated stimuli to induce spontaneous germinal centers (24). Under these circumstances, IFNγ signaling in B cells results in increased IL-6 and IFNγ production which drives auto-immunity (20, 21). In contrast, our *ex vivo* stimulated B cells lacked additional stimuli and upregulated expression of PD-L1 and TGF-β, while we did not see any effects on IL-6 and IFNγ. We further showed that IFNγ-B cells express high levels of CCR7 with minimal changes in CXCR5 and Ebi-2. This expression profile is in line with a previous study that demonstrated that MZ B cells from *ldlr*^−/−^ mice interact with pre-T_FH_ cells at the T-B cell border in response to a high-cholesterol diet (13). Moreover, we show that *ex vivo* IFNγ stimulation generates a PD-L1^hi^ B cell pool containing enhanced MZ and GC B cells, while pro-atherogenic FO B cells are decreased. During our atherosclerosis study, we indeed found that adoptively transferred IFNγ-B cells showed the characteristics of B cells that reside in or near the germinal center.

Similar to flow-sorted PD-L1^hi^ B cells (16), we next showed that IFNγ-B cells were able to inhibit T_FH_ cell numbers *in vitro* and in our short *in vivo* experiment. Notably, at the point of sacrifice in our atherosclerosis experiment we did not observe restriction of T_FH_ cells by IFNγ-B cells, but we did demonstrate that IFNγ-B cells were able to promote anti-inflammatory CD4^+^ T cells. This corresponds with the effects found in experimental autoimmune encephalomyelitis using PD-L1^hi^ B cells, which restricted Th1 and Th17 differentiation (16). Along this line, we found that IFNγ-B cells resulted in decreased Th17 cells and a significant increase in atheroprotective Tregs and IL-10 production of CD4^+^ T cells. Interestingly, it has been reported that during atherosclerosis development there is a plasticity between Treg cells and T_FH_ cells and disturbances of this delicate balance greatly affected atherosclerosis development (14). We did not directly investigate this plasticity in our study, but our work shows that IFNγ-B cells are able to inhibit T_FH_ cells and increase Treg cells.

Furthermore, we demonstrated atheroprotective effects of IFNγ-B cells that express high levels of PD-L1.The observed reduction in atherosclerosis upon adoptive transfer of IFNγ-B cells was accompanied by changes in plaque morphology (relatively more macrophages, reduced collagen), indicating a more initial plaque phenotype. Reduced collagen content did not coincide with alterations in overall VSMCs content. Although we cannot specifically determine collagen production rate by SMCs locally in the plaque, the lack of difference in SMC content suggests that the difference in collagen content that we observed in the plaque is not explained by the amount of SMCs. Although we did not investigate this in our study, the reduced collagen content in IFNγ-B cell treated mice may also be attributed to an increase in collagen degradation through the production of matrix metalloproteinases by the macrophages in the plaque, which were relatively increased upon IFNγ-B cell transfer. The atheroprotective effect of PD-L1^hi^ B cells is in line with a general protective role of the PD-L1/PD-1 axis in atherosclerosis. Mice deficient in both PD-L1 and PD-L2 show increased atherosclerosis (25). Similarly, PD-1 knockout mice or mice treated with a PD-1 blocking antibody developed exacerbated atherosclerosis (26, 27), whereas stimulation of PD-1 signaling reduces atherosclerosis (28). Our data is further supported by previous studies that demonstrated the proatherogenic role of T_FH_ cells (12–14).

However, due to the pleiotropic nature of IFNγ, other factors besides PD-L1 could also have contributed to our findings. Indeed, the observed increase in TGF-β expression could have contributed to both the T_FH_ inhibition and Treg induction, since TGF-β signaling is known to prevent T_FH_ cell accumulation and can promote Tregs (29). In addition, there have been a large number of studies with adoptive transfer of B cells expressing TGF-β that reported immune tolerance in autoimmune mouse models (30–33). The majority of these studies reported Treg induction, which is in line with our findings of increased Tregs and IL-10 production after adoptive transfer of IFNγ-B cells. PD-L1 is also known to be essential for the induction of Treg cells (34), the Treg accumulation could thus be a combined effect of increased TGF-β and PD-L1 expression by IFNγ-B cells. Since the atheroprotective effects of Treg cells is well characterized (10, 35), the observed Treg induction undoubtedly contributed to the reduced atherosclerosis found with adoptive transfer of IFNγ-B cells. Moreover, we show that *ex vivo* IFNγ stimulation generates a pool of PD-L1^hi^ B cells which contains reduced FO B cells. FO B cells can contribute to atherosclerosis progression (15), and despite the increased co-inhibitory PD-L1 expression following IFNγ exposure, we cannot exclude that a decrease in FO B cells in the adoptively transferred IFNγ-stimulated B cells also contributed to the observed anti-atherogenic effect.

In conclusion, this study uncovers a new role for *ex vivo* stimulation of B cells with IFNγ for the induction of atheroprotective B cells. IFNγ-B cells show the genetic makeup of GC B cells with increased expression of PD-L1 and TGF-β and effectively inhibit T_FH_ cells *in vitro* and *in vivo* and ameliorate atherosclerosis development in *apoE*^−/−^ mice. These results further emphasize the proatherogenic role of the T_FH_–GC B axis and provide a novel way to regulate this axis.

## Data Availability Statement

The raw data supporting the conclusions of this article will be made available by the authors, without undue reservation.

## Ethics Statement

The animal study was reviewed and approved by Leiden University Animal Ethics Committee.

## Author Contributions

HD, JM, GV, JK, and AF contributed to the conception and design of the study. HD, JM, JA, FS, BS, MKi, MKr, IB, GV, and AF carried out the experiments and acquired the data. HD and JM performed the data analysis. HD, JM, and AF wrote the manuscript. CB, JK, and AF provided critical feedback to the manuscript. JK and AF supervised the project. All authors read and approved the submitted version.

## Funding

This work was supported by the European Union's Seventh Framework [Grant Number 603131], by contributions from Academic and SME/industrial partners, supported by the Dutch Heart Foundation [Grant Numbers 2016T008 and 2018T051 to AF] and by the European Research Area Network (ERA-CVD B-eatATHERO consortium) supported by the Dutch Heart Foundation [2019T107 to AF]. IB is an Established Investigator of the Dutch Heart Foundation [2019T067].

## Conflict of Interest

The authors declare that the research was conducted in the absence of any commercial or financial relationships that could be construed as a potential conflict of interest.

## Publisher's Note

All claims expressed in this article are solely those of the authors and do not necessarily represent those of their affiliated organizations, or those of the publisher, the editors and the reviewers. Any product that may be evaluated in this article, or claim that may be made by its manufacturer, is not guaranteed or endorsed by the publisher.
