# Supplementary material for: IFNγ-Stimulated B Cells Inhibit T Follicular Helper Cells and Protect Against Atherosclerosis
Source: Front Cardiovasc Med. 2022 Feb 2;9:781436. doi: 10.3389/fcvm.2022.781436 (PMC8847680; doi:10.3389/fcvm.2022.781436)
Supplement: Supplementary file 1 [file Data_Sheet_1.docx]

Supplementary Material

IFNγ-stimulated B cells inhibit T follicular helper cells

and protect against atherosclerosis

H. Douna^1#^, J. de Mol^1#^, J. Amersfoort^1^, F. H. Schaftenaar^1^, M. G. Kiss^2,3^, B.E. Suur^1^, M. J. Kroner^1^, C. J. Binder^2,3^, I. Bot^1^, G.H.M. van Puijvelde^1^, J. Kuiper^1^, A.C. Foks*^1^

^1^ Division of BioTherapeutics, LACDR, Leiden University, Leiden, The Netherlands

^2^ Department of Laboratory Medicine, Medical University of Vienna, Vienna, Austria

^3^ CeMM Research Center for Molecular Medicine of the Austrian Academy of Sciences, Vienna, Austria

^#^ Authors contributed equally.

# Supplementary Figures and Tables


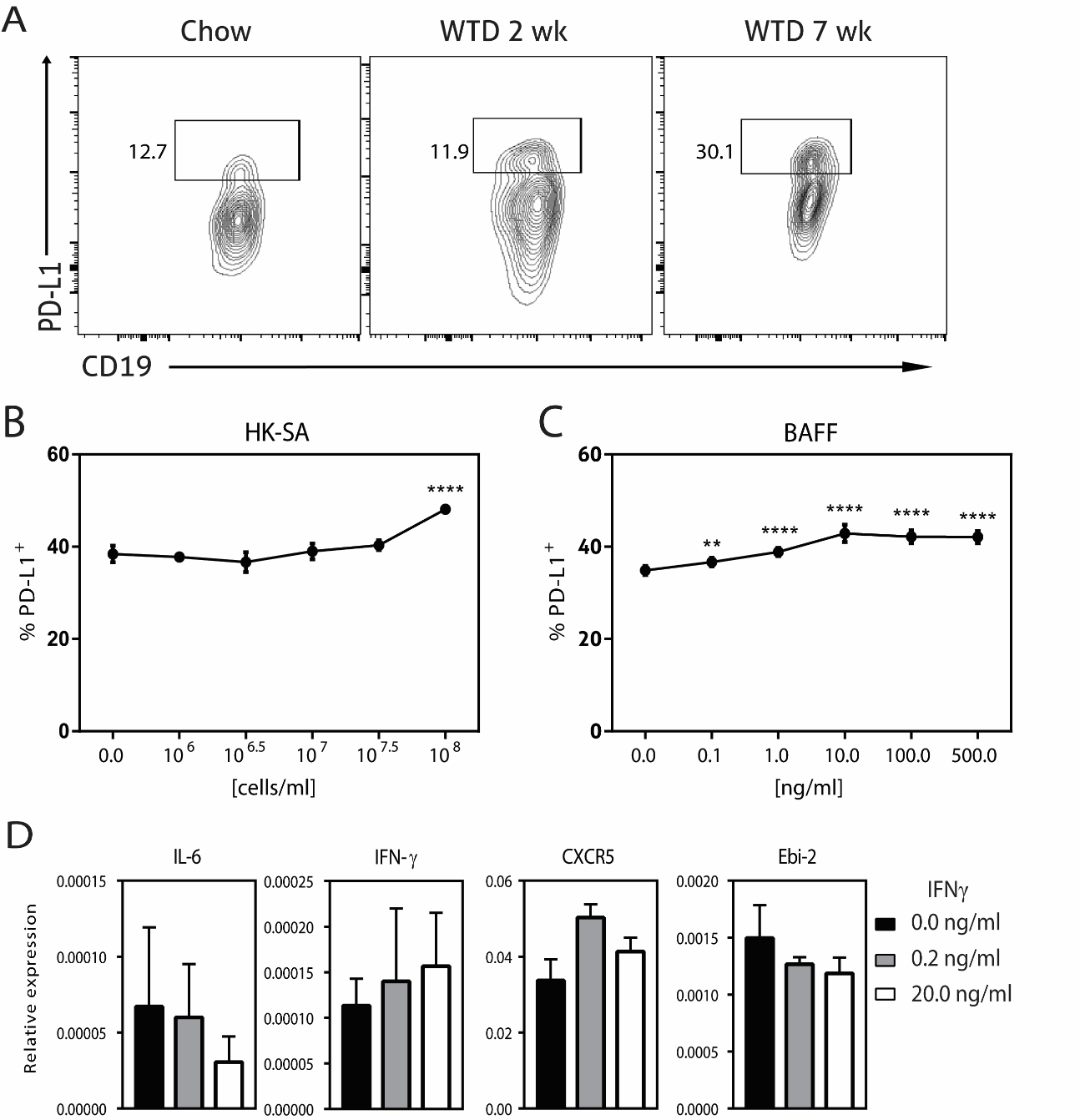


**Supplementary Figure 1. PD-L1 expression on B cells**. **(A)** Representative flow cytometry contour plots of PD-L1^hi^ expressing CD19^+^ B cells in *apoE^-/-^* mice fed a chow diet or a WTD for 2 or 7 weeks. CD19^+^ B cells were isolated from C57BL/6 mice and stimulated for 24 hours with different doses of **(B)** heat killed *S. Aureus* (HK-SA) or **(C)** B cell activating factor (BAFF) after which PD-L1 expression was measured with flow cytometry. **(D)** CD19^+^ B cells were unstimulated or stimulated with 0.2 ng/ml or 20.0 ng/ml IFNγ for 24 hours, after which mRNA expression of depicted genes was assessed using qPCR. Data are analyzed with a One-Way ANOVA and shown as mean ± SEM (** p<0.01, ****p<.00001). n=3/group.


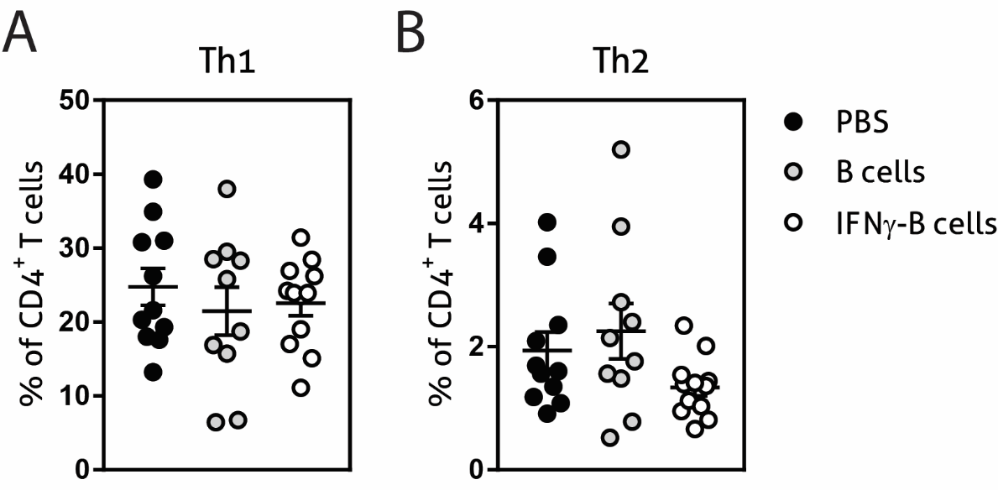


**Supplementary Figure 2. Effects of adoptive transfer of IFNγ-B cells on Th1 and Th2 cells*.*** *ApoE^-/-^* mice were fed a Western type diet for 7 weeks. After 2 weeks they received a perivascular collar and were treated with PBS, freshly isolated B cells (B cells) or B cells stimulated with 20.0 ng/ml IFNγ for 24 hours (IFNγ-B cells). Mice received a total of three injections and injections were spaced every two weeks. After 7 weeks, mice were sacrificed and spleens were analyzed with flow cytometry for **(A)** Th1 cells (T-bet^+^) and **(B)** Th2 cells (Gata-3^+^). Data are shown as mean ± SEM. n=10-12/group.


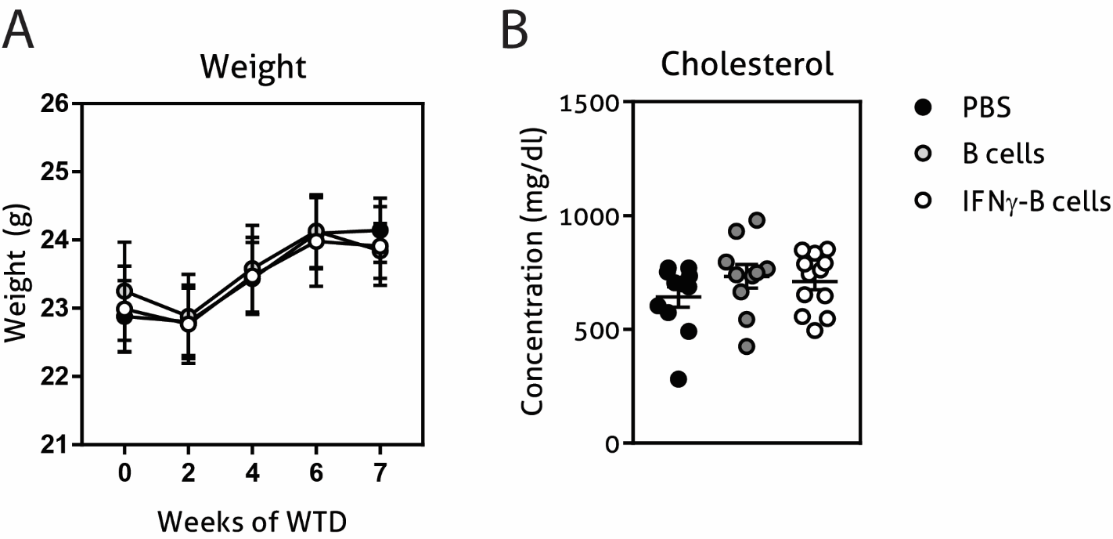


**Supplementary Figure 3. Weight and cholesterol levels.** *ApoE^-/-^* mice were fed a Western type diet for 7 weeks. After 2 weeks they received a perivascular collar and were treated with PBS, freshly isolated B cells (B cells) or B cells stimulated with 20.0 ng/ml IFNγ for 24 hours (IFNγ-B cells). Mice received a total of three injections and injections were spaced every two weeks. **(A)** Weight was assessed during the experiment and **(B)** and cholesterol serum levels were analyzed at the end of the experiment. Data are shown as mean ± SEM. n=10-12/group.


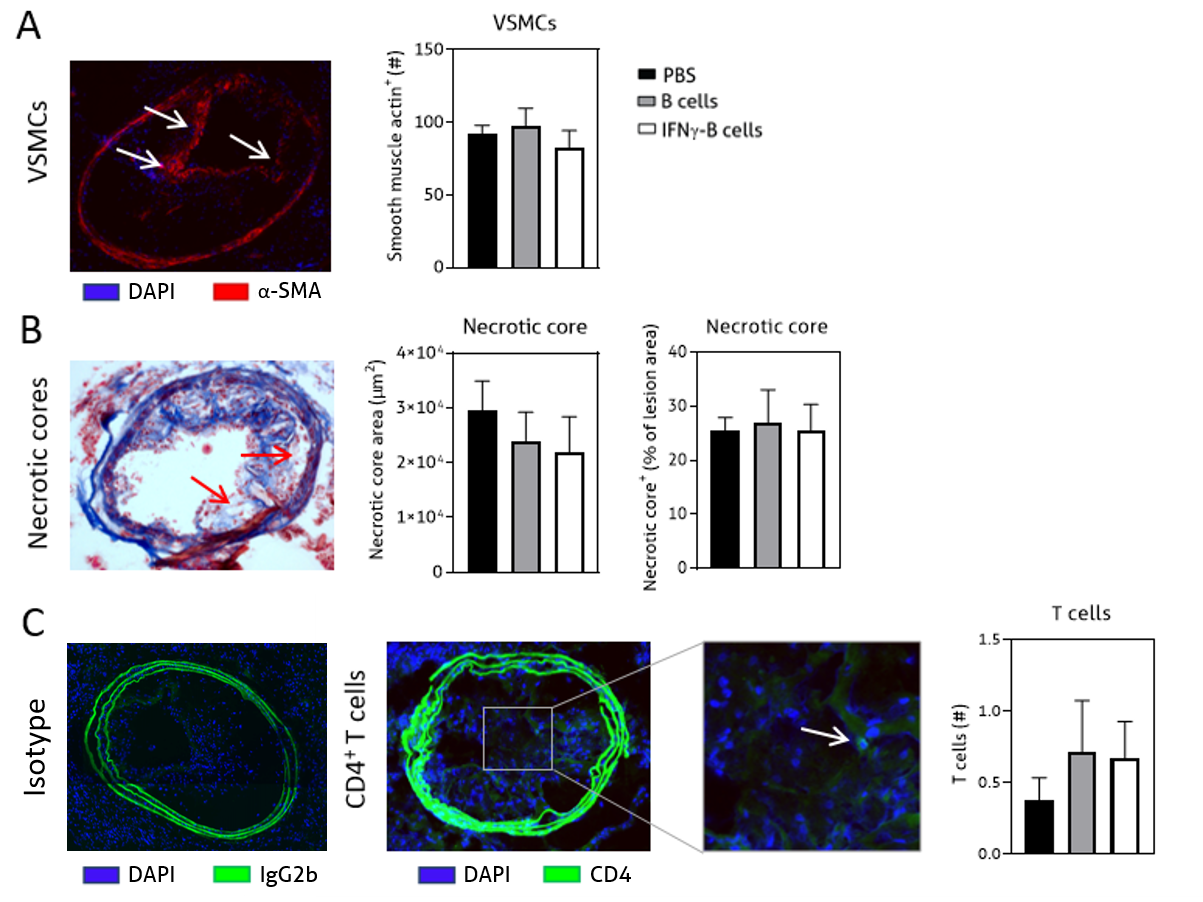


**Supplementary Figure 4. Adoptive transfer of IFNγ-B does not affect VSMCs, necrotic core size and T cell infiltration in atherosclerotic plaques.** *ApoE^-/-^* mice were fed a Western type diet for 7 weeks. After 2 weeks they received a perivascular collar and were treated with PBS, freshly isolated B cells (B cells) or B cells stimulated with 20.0 ng/ml IFNγ for 24 hours (IFNγ-B cells). Mice received a total of three injections and injections were spaced every two weeks. Cryosections of the right carotid artery containing the largest lesions were used to determine **(A)** VSMCs content (red) using an α-smooth muscle actin staining, **(B)** necrotic core size as by selecting acellular areas in the Masson’s trichrome stained sections and calculated as percentage of the total plaque area, and **(C)** CD4^+^ T cell infiltration using a CD4 antibody (green, right), with rat IgG2b isotype (green, left) as control. Data are shown as mean ± SEM. n=5-12/group.

**Supplementary table 1. qPCR primers.**

**Supplementary table 2. Antibodies for flow cytometry.**
